# The costs of removing the unsanctioned import of marine plastic litter to small island states

**DOI:** 10.1038/s41598-020-71444-6

**Published:** 2020-09-10

**Authors:** April J. Burt, Jeremy Raguain, Cheryl Sanchez, Jude Brice, Frauke Fleischer-Dogley, Rebecca Goldberg, Sheena Talma, Martyna Syposz, Josephine Mahony, Jake Letori, Christina Quanz, Sam Ramkalawan, Craig Francourt, Ivan Capricieuse, Ash Antao, Kalsey Belle, Thomas Zillhardt, Jessica Moumou, Marvin Roseline, Joel Bonne, Ronny Marie, Edward Constance, Jilani Suleman, Lindsay A. Turnbull

**Affiliations:** 1 Department of Plant Sciences, Oxford University, South Parks Road, Oxford, OX1 3RB UK; 2 Seychelles Islands Foundation, La Ciotat Building, Mont Fleuri, Victoria, Mahé, Seychelles; 3 Department of Zoology, Oxford University, 1a Mansfield Rd, Oxford, OX1 3SZ UK; 4 Department of Geography, Oxford University, South Parks Road, Oxford, OX1 3QY UK; 5 Department of Materials Science, Oxford University, 21 Banbury Rd, Oxford, OX2 6HT UK

**Keywords:** Environmental impact, Environmental economics

## Abstract

Small island states receive unprecedented amounts of the world’s plastic waste. In March 2019, we removed as much plastic litter as possible from Aldabra Atoll, a remote UNESCO World Heritage Site, and estimated the money and effort required to remove the remaining debris. We removed 25 tonnes at a cost of $224,537, which equates to around $10,000 per day of clean-up operations or $8,900 per tonne of litter. We estimate that 513 tonnes (95% CI 212–814) remains on Aldabra, the largest accumulation reported for any single island. We calculate that removing it will cost approximately $4.68 million and require 18,000 person-hours of labour. By weight, the composition is dominated by litter from the regional fishing industry (83%) and flip-flops from further afield (7%). Given the serious detrimental effects of plastic litter on marine ecosystems, we conclude that clean-up efforts are a vital management action for islands like Aldabra, despite the high financial cost and should be integrated alongside policies directed at ‘turning off the tap’. We recommend that international funding be made available for such efforts, especially considering the transboundary nature of both the marine plastic litter problem and the ecosystem services provided by biodiversity-rich islands.

## Introduction

In the last decade the world has woken up to the extent and impact of plastic pollution in the oceans^1^. Approximately 6,300 million metric tonnes of plastic waste has now been generated worldwide, and because collection and recycling facilities are unavailable or inadequate in most countries, up to 12.7 million tonnes is estimated to enter the oceans annually^2^. This figure is likely to remain high for the foreseeable future, despite global efforts to reduce single-use plastics^3–7^. Although these numbers provide an overview of the global problem, they do not reveal the full environmental and financial impact on countries that unwillingly import this waste, which arrives on their shores every day. While source interventions are being discussed and slowly implemented^8^, the down-stream accumulation is taking its toll at both local and national level and cannot be ignored, especially in light of the accumulating evidence of the detrimental effects of plastic, microplastics and plastic leachate on species and ecosystems^9–15^.

The Republic of Seychelles is one such nation: a small island state, with 155 islands stretching over 1.4 million km^2^ of the Southwest Indian Ocean and home to only 98,000 people^16^. One of the largest and most remote marine protected areas within Seychelles is Aldabra Atoll, among the largest raised coral atolls in the world and a UNESCO World Heritage Site. Aldabra is an iconic site, both within Seychelles and globally; heavily protected, it acts as an important benchmark for the impacts of global environmental change. It is home to the last remaining population of Indian Ocean giant tortoises^17^ (*Aldabrachelys gigantea*) and provides one of the largest nesting sites for endangered green turtles (*Chelonia mydas*)^18^ in the western Indian Ocean. The atoll supports large colonies of seabirds^19^, 11 endemic land-bird species/sub-species^20^ and the biomass of fish and sharks in its extensive coral reef and mangrove ecosystems is among the highest in the Indian Ocean^21^. The ecosystem services that Aldabra and other Seychelles island ecosystems provide are vital to human health and prosperity in the Seychelles and across the wider region but have already been subject to intense pressure from invasive alien species^22,23^, habitat loss^24^and climate change^25^. To maximise the resilience of these ecosystems to climate change, especially in the marine ecosystems adjacent to islands, governments and managers must ensure the ecosystems are as healthy as possible. The arrival and accumulation of marine plastic litter is both an un-quantified threat to these ecosystems and yet another burden to the organisations and government departments tasked with managing and conserving sites and species^26^.

**Figure 1:**
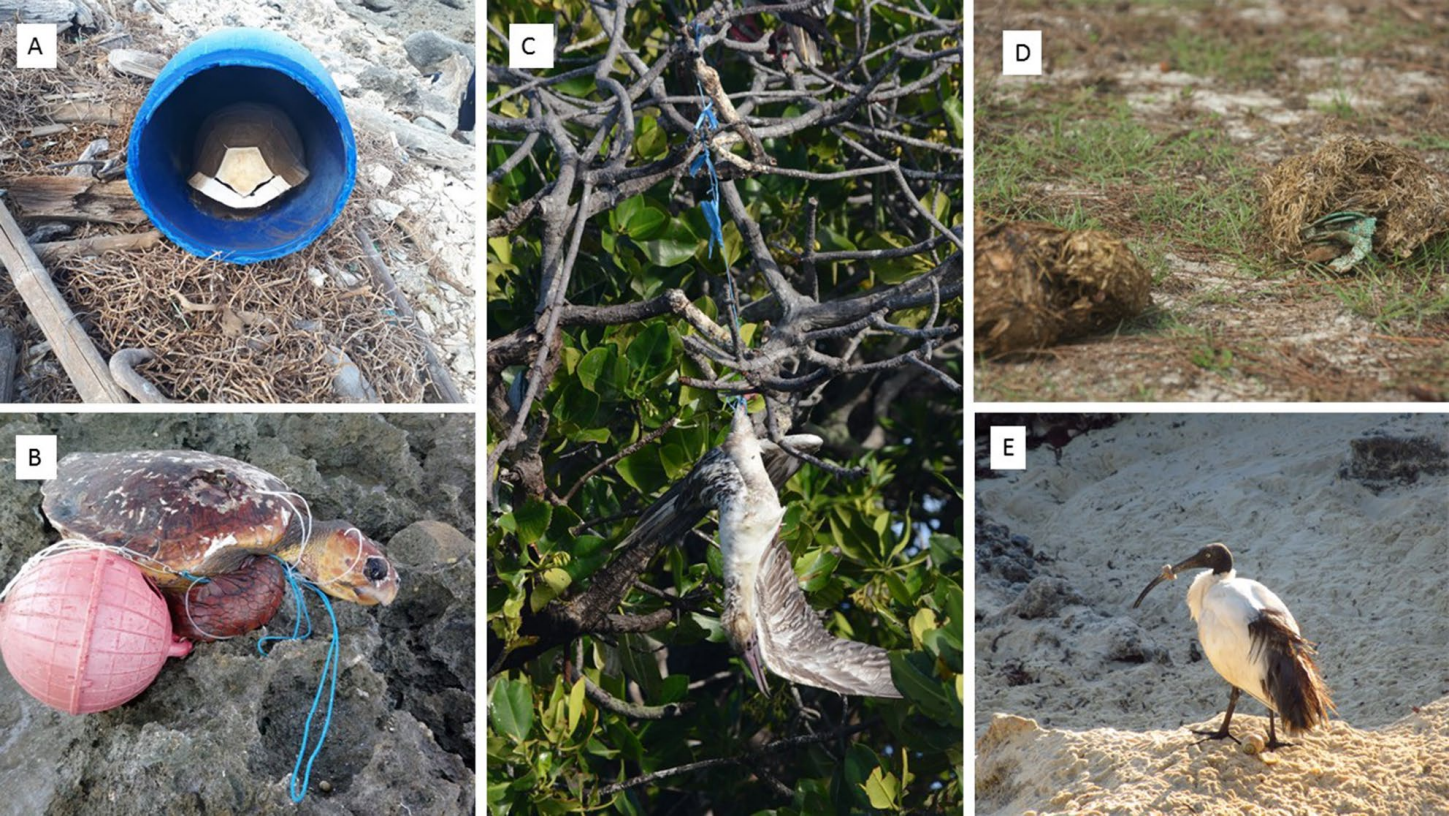
Impacts and threats from marine plastic litter to Aldabra’s ecosystem: (**A**) dead giant tortoise (Aldabrachelys gigantea) inside plastic barrel; (**B**) dead loggerhead turtle (Caretta caretta)—one of many species to migrate past the atoll—entangled in fishing gear and washed up on the coast; (**C**) dead red-footed booby (Sula sula) entangled in plastic fishing-line; (**D**) giant tortoise faeces with partial flip-flop—evidence that tortoises are ingesting plastic; (**E**) sacred ibis (Threskiornis bernieri)—one of two individuals observed by the team with its beak trapped shut by a ring of plastic, possibly an eroded cap from a PET bottle.

**Figure 2:**
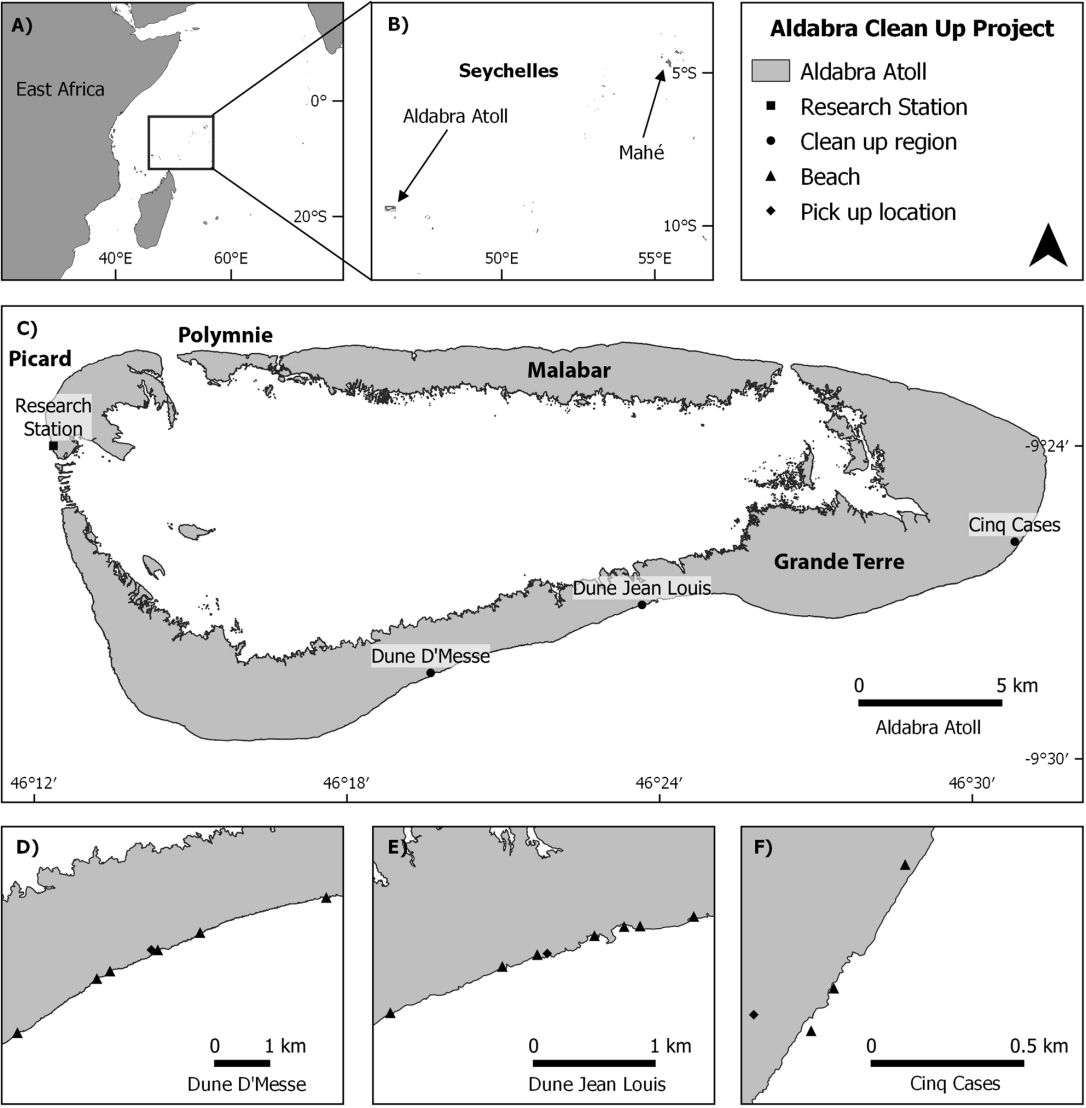
The location of Aldabra Atoll and the clean-up operations: (**A**) Aldabra in the Indian Ocean; (**B**) Aldabra’s proximity to the main Seychelles Islands; (**C**) Aldabra atoll and the clean-up regions in relation to the research settlement; (**D**) Dune D’Messe camp and beach locations; (**E**) Dune Jean Louis camp and beach locations and (**F**) Cinq Cases camp and beach locations. QGIS Development Team V3.14, 2020. https://qgis.osgeo.org.

The increasing accumulations of marine plastic litter along Aldabra’s coastlines together with direct entanglement, ingestion and injuries to a wide range of species in discarded fishing gear (Fig. 1) is unacceptable at this iconic site, as it would be if the same amount of plastic rubbish had been allowed to accumulate in one of the world’s great museums or art galleries. We (The Seychelles Islands Foundation (SIF) who manage the site) initiated a clean-up project in 2019 with the twin aims of: (1) documenting the extent and nature of the plastic problem and, critically; (2) attempting to remove as much accumulated plastic litter as possible from the south coast of Grande Terre Island (Fig. 2), the largest of four islands that comprise the atoll. While previous studies have catalogued the extent of marine plastic pollution on remote islands around the world^27–29^, these expeditions have never made a serious attempt to remove it, nor have they documented the associated costs. Quantifying the resources needed for removal efforts is critical to estimate the financial burden on small island states like Seychelles to manage marine plastic litter, and to allow such nations to adapt and plan accordingly.

With long-term management in mind, we estimated the total amounts and types of marine plastic litter found on Aldabra to quantify the source of the problem and to estimate the cost of a clean-up effort for the entire atoll. To do this we recorded all costs associated with the clean-up, and the effort required to do so, by timing clean-up sessions and estimating the amount of litter collected per person per unit time. The waste collected was weighed and categorised and additional surveys were conducted in each coastal habitat type to estimate the total remaining marine plastic litter on Aldabra and its composition. The composition data was used to determine the main sources of litter arriving on Aldabra.

## Results

### Collected waste

During the clean-up a total of 26.4 tonnes of marine plastic litter was collected from the coastal areas of Grande Terre, but only 25.7 tonnes (approximately 250 m^3^) could be removed from the shore to the cargo vessel and transported back to Mahé. The remaining 0.7 tonnes had to be left on Aldabra due to unsafe sea conditions that made removal from one of the smaller collection points (Cinq Cases) impossible.

Of the litter collected and removed from Aldabra, the largest component by weight was fishing-related items (buoys, nets, FADS and ropes) which collectively made up 60% (15.8 tonnes) of the total, followed by plastic shoes (mostly flip-flops), which made up 24% (6 tonnes) of the total (Figure S1). During the entire five-week clean-up we estimate that 60,000 individual flip-flops were collected.

### Estimations of remaining litter

**Figure 3:**
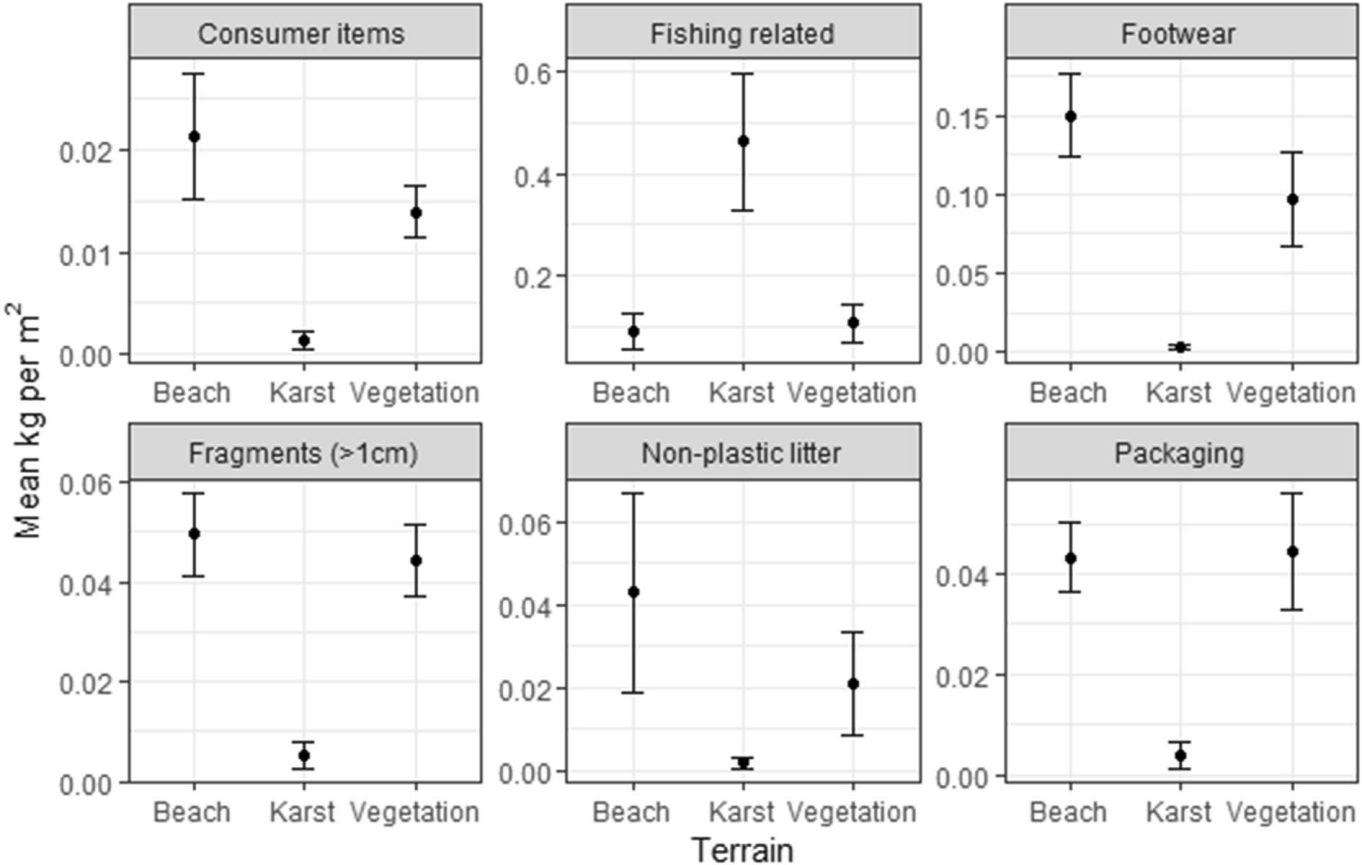
The amounts of plastic waste in each of six categories recorded in twenty transects in each of the three main terrain types (60 transects in total). Error bars show one standard error. The scale on the y-axis varies among plots.

The transects revealed that different habitat types accumulate different relative amounts of each type of litter (Fig. 3; Table S1; F_10,285_ = 14.96; *P* < 0.001): the proportion of heavy fishing ropes was highest on the limestone karst, while PET bottles and other light consumer items were collected in greater quantities on the beach and vegetation terrain. Based on the raw data, we estimate the total amount of accumulated litter on Grande Terre to be 513.4 (95% CI 212–814) tonnes. The estimated composition of this litter by weight is 83% (426 tonnes) fishing related, 7% (36 tonnes) footwear, 4% (20.5 tonnes) fragments, 3% (15.4 tonnes) packaging, 2% (10 tonnes) other types of litter and 1% (5 tonnes) consumer items (Figure S1). Note, that the estimated composition of the litter remaining on Aldabra is different to the composition of the litter collected and removed during the expedition. This is because the expedition preferentially targeted beaches, rather than karst, where the proportion of fishing gear is lower.

### Effort and future clean-up

The mean collection rate calculated from 40 clean-up sessions totaling 980 collection hours was 28 kg (SE = 1.84) per hour per person, meaning that it requires 35.7 person-hours to remove 1 tonne of litter. We therefore estimate that it would take a team of 12 people approximately 191 (73 to 303) days (assuming two four-hour sessions per day) to collect the remaining 513 tonnes of marine plastic litter from the largest island of Grande Terre. It took four days to move 26 tonnes of waste from shore to ship, so we estimate that 82 days would be necessary to move all the remaining waste from shore to ship. As the total cost of the five-week clean-up was $224, 537, we estimate that a full clean-up would cost approximately $4.68 million (95% CI: $1.95 million – $7.28 million) (Table 1). This is around $10,000 per day of clean-up operations or $8,900 per tonne.

**Table 1:** Summary of the costs involved in the 34-day clean-up.

Action	Cost in Seychelles Rupee	Cost in GB Pound	Cost in US Dollar	Average daily cost in US Dollar
Equipment (3,000 sacks and 825 slings)	SCR 184,350	£ 10,323	$ 13,457	$ 395
Team Food (12 people)	SCR 64,438.9	£ 3,678.83	$ 4,703	$ 138
Team Transport	SCR 669,313	£ 38,211	$ 49,504	Not included
Cargo vessel (18 days in total)	SCR 2,057,318	£ 117,482	$ 152,163	$ 8,454
Small boats fuel (4 days of pick-up shuttles)	SCR 30,000	£ 1,694	$ 2,191	$ 547
SIF staff (based on average staff salary, daily rate for ten staff over 4 days for pick-up)	SCR 20,000	£ 1,129	$ 1,461	$ 365
Cost of coastguard deployment*	SCR 1,100,000	£ 62,111	$ 80,372	Not included
Total	SCR 3,025,420	£ 172,722	$232,588	$ 9,899

*This was not covered by project costs but by the Seychelles Government. The coastguard deployment would not necessarily be essential in future operations, if sufficient additional staff were present, therefore the cost has not been included here.

### Origin and type of litter

Of the 470 PET bottles sampled, 40% were originally used for water, 8% for soft drinks (of which 62.5% carried labels from the Coca-Cola Company) and 52% were unidentifiable. Of the 470 sampled bottles, 45 had intact labels where the manufacturing country was identified (Fig. S2). Of these, the majority were from China (21), with others coming from Indonesia (6), Thailand (4), Malaysia (3), India (3), Singapore (2) and South Africa (1).

Of the fishing floats sampled, 28% (*n* = 50) had ‘Made in Taiwan’ inscribed on them. The remaining floats were branded (e.g. 11 were Blue Dolphin, four were Blue Fin) but had no clear origin. Of the fish-aggregation devices or FADs (*n* = 13), seven had clearly decipherable identification codes and all came from purse-seine vessels registered to fish in the Seychelles Exclusive Economic Zone; five were from Seychellois vessels; one was Spanish and one French.

## Discussion

The clean-up removed around 25 tonnes of marine plastic litter from Aldabra – around 5% of the estimated total—at a cost of $224,538 and 980 person-hours of effort. By combining information on amounts and types of litter with the effort required to remove it, we estimate a total projected cost of around $1.95–$7.28 million to clean up the entire south island, where the vast majority of the litter lies. This eyewatering price-tag makes the economic burden of the unsanctioned import of plastic litter on small island states abundantly clear.

Our estimate of ~ 500 tonnes (within a total area of 1,166,508 m^2^; Table S1) of marine plastic litter on Aldabra is probably conservative for a number of reasons: (1) it only includes the litter on Grande Terre, the largest island of Aldabra; (2) it does not include buried beach debris, which is substantial on some beaches, due to wind action and digging by nesting turtles. Nevertheless, our estimate is the highest recorded for any one island worldwide (0.44 kg per m^2^). In comparison, the estimate for Henderson Island, another World Heritage Site, is 18 tonnes (ca. 0.18 kg per m^2^)^27^, and 238 tonnes on the Cocos Island group (ca. 0.41 kg per m^2^)^28^ using a similar methodology, although these studies also include micro-debris (2–5 mm) and buried debris (to 10 cm depth). The amount of plastic litter accumulating on Aldabra seems to be typical of the situation on a number of other Seychelles islands^29,30^, although no estimates of total waste accumulation have been made. All such islands have high conservation value due to the marine ecosystems they support^21^.

The challenges and subsequent costs of removing 25 tonnes of litter from Aldabra are likewise representative of the likely costs involved in a significant clean-up of a remote tropical island anywhere in the world. The challenges include the distance from the mainland and the lack of facilities to support large-scale clean-up operations such as team accommodation, transport, food and water; not to mention the inherent danger in manually moving large fishing nets into small boats in considerable swell (Fig. S3). The challenges at Aldabra may be more extreme than at other islands due to the remote location, although some other islands, e.g. Henderson Island, are equally remote, but have no resident team, and hence detailed knowledge of currents and landing sights is limited, making clean-ups potentially harder. The estimated total project cost is conservative because this figure does not include in-kind contributions from SIF staff, who managed the planning and logistics for the clean-up, or the cost of the Seychelles coastguard vessel. A recent study estimated that the annual cost of marine plastic litter in terms of reduced natural capital of the affected ecosystems lies somewhere between $3,300 and $33,000 per tonne^31^; but this is only the cost of inaction, while the cost of active clean-up operations is rarely reported. In our case, the clean-up cost per tonne is around $8,900 and so, removing most or all of the accumulated litter from the largest island will likely cost around $1.95 million–$7.28 million—well beyond the capacity of non-profit organisations like SIF.

Understanding where this waste originated is a vital step in allocating accountability, to galvanise reduction at the source and potentially pinpoint where clean-up costs could be generated. It has been widely reported that approximately 80% of marine plastic litter originates on land, while the remaining 20% derives from marine sources^12,32^, half of this being directly related to the fishing industry. Our results, however, suggest the opposite: 60% of the litter that we collected during the clean-up – and 83% of the estimated total remaining on Aldabra—was fishing-related. This high proportion of fishing-industry waste is comparable to that found in the Great Pacific Garbage Patch (52%)^33^, on islands in the Atlantic Ocean (> 40%)^34^, on Australia’s northern shores (63%)^35^ and across the five sub-tropical gyres (58.3%)^36^. Elsewhere in the Indian Ocean, the proportion of litter from the fishing industry is much lower; for example, it constitutes 7.7% of the litter on Henderson Island, 1.5% of the litter on Cocos Keeling islands^27,28^ and just 2% of that on nearby Alphonse island^29^. These large differences in the proportion of fishing gear at sites within the region and the high proportion of fishing gear retrieved during this clean-up suggest that Aldabra is a hotspot for accumulation. The arrival of particular types of waste may be heavily influenced by proximity to sources of litter, and/or by the location of islands in particular ocean currents, so will inevitably differ from place to place.

The economy of Seychelles is highly dependent on fishing, which directly and indirectly employs around 10% of the population. Industrial tuna fishing – mostly based on yellowfin (*Thunnus albacares*), skipjack (*Katsuwonus pelamis*) and bigeye tuna (*Thunnus obesus*) – is one of the most important sources of foreign currency earnings^37^. The industrial fishery that operates within the Seychelles Exclusive Economic Zone (EEZ) is composed of licensed vessels, both Seychelles and foreign registered, that fall under two main categories: purse-seiners (mainly EU owned vessels) and longliners (mainly Taiwanese and Chinese vessels). Since the early 1990′s, purse-seiners have been mainly operating using fish aggregative devices (FADs). The number of FADs that each ship can maintain is restricted, but because they float freely, they can enter marine protected areas, where the boats are not permitted to follow them. In an assessment of drifting FADs within the Indian Ocean, 9.9% of deployments by purse-seine fisheries were estimated to end with a beaching event, suggesting that 1,500–2,000 may be lost onshore each year in places like Maldives, the Chagos Archipelago, and Seychelles^38^. Of the 13 FADs collected during the clean-up expedition, we were able to trace the origin of seven, all to purse-seiners that are authorised to fish in the Seychelles EEZ. A recent report for the Indian Ocean Tuna Commission investigated 214 individual FADs that had arrived on or entered into near-shore waters of a number of islands in Seychelles: 76% of the FADs were from Spanish owned or flagged vessels, licensed to fish in Seychelles^39^. These results are alarming: first because it shows that waste generated by the fishing industry within Seychelles is polluting island ecosystems within the same nation state; second, if the fishing industry is the major contributor to marine plastic litter in the region, then it is almost certainly having indirect negative impacts on the fish communities it needs to sustain^9^. Our results also indicate that the amount of litter estimated to be circulating in the region is greatly underestimated and highlights the flaws of using sweeping global estimates within a local context. In 1990 the Seychelles acceded to the MARPOL convention and its mandatory Annexes I (Prevention of Pollution by Oil) and II (Control of Pollution by Noxious Liquid Substances in Bulk). Largely in response to this clean-up and other similar efforts by local conservation organisations, which have already received widespread local publicity, the Seychelles Government decided to accede to the remaining MARPOL annexes in 2019; most importantly it acceded to Annex V- *Prevention of Pollution by Garbage from Ships*, demonstrating that highlighting the problem can lead to political action by concerned governments.

Previous studies of island litter accumulation have focused on quantifying the number of individual items found: 414 million on Cocos Island Group^28^ and 37.7 million on Henderson Island^27^; however, we chose not to do this, focussing instead on the total weight and time required to collect and remove it, as these are likely to be more important for managers planning clean-ups. We deliberately excluded fragments below 1 cm diameter and wish to highlight the impossibility of removing such small fragments as part of clean-up efforts in such remote locations unless new technology become available. Although small items generate microplastics, we suggest that removing larger items before they degrade into tiny particles is the best way to reduce the amount of microplastics entering the local ecosystem.

The six tonnes of flip-flops collected during the expedition equates to approximately 60,000 individual flip-flops, and the estimated 37.2 tonnes of footwear remaining on Aldabra approximates to 370,200 individual flip-flops. Unlike the litter from the fishing industry, it is less likely that the main source of these is Seychelles: there are simply too many to imagine that they originate from a population of 98,000 people. The flip-flop phenomenon is not unique to Aldabra, and other studies throughout the region have reported large numbers and speculated about their origins^29,30,40^. Duhec et al.^29^ mention that flip-flops are the world’s simplest, cheapest and most popular shoe^41^, especially in countries that fringe the Indian Ocean, where waste management is extremely poor.

While we were unable to determine the source of individual flip-flops, as most were either too degraded or non-branded, we recorded the country of origin of a sample of PET drink bottles. Perhaps unsurprisingly, given its size and population density, 47% of plastic bottles found on Aldabra with intact labels were from China. This is similar to findings from islands in the Southern Atlantic Ocean^42^, and on Alphonse Island in Seychelles, where more than 75% of labelled items originated in Southeast Asia (mainly Indonesia and Thailand) and 13% originated in East Asia (mainly China)^29^. In both cases, the original studies suggested that the intact labels might indicate the source of the bottles was dumping from ships. Only a small proportion of bottles found on Aldabra had intact labels, so we suggest that this hypothesis does not hold for Aldabra. Those found with labels clearly indicate their origin and these data corroborate findings from previous studies showing that globally, the largest input of land-based marine plastic pollution is countries in Asia^2,43^.

Regardless of where the waste originated, the people and government of Seychelles are left to manage and dispose of it. The current waste management system in Seychelles is restricted to landfills and several small-scale initiatives, such as collections of aluminium cans and PET bottles^44^, which are then exported for recycling. At present, there are no in-country recycling options for any plastics^45^ and marine-degraded plastic requires specialist recycling. The relatively small amount of waste we returned to Mahé spent six months in a holding site while we attempted to find local re-use and re-purposing solutions. In total, 9.2% of the litter was re-used by conservation organisations, local schools and artists, but these are not long-term pipelines for waste. Recycling solutions exist elsewhere in the world to enable a circular economy for the majority of plastic types (Table S2), although solutions may not exist at appropriate scales. One difficulty is that the costs of collecting and moving waste from remote sites are likely to make any solutions economically unviable, but this requires further analysis. For example, if the resulting products can be branded as originating in a UNESCO World Heritage Site, it’s possible that some value might be added; prior to the COVID-19 epidemic, Seychelles had a thriving high-end tourist industry^46^ and there may be a market for high-quality goods made from recycled materials to sell to tourists locally**.**

It is reasonable to ask whether, given their extremely high cost, clean-up efforts should take place at all. However, we are not predicted to reach global “peak waste” before 2100^47^and it’s simply unimaginable that plastic litter be allowed to accumulate on Aldabra at current rates for another 80 years. The arrival of tonnes of plastic litter compromises the integrity of this near-pristine system and potentially increases its vulnerability to climate change; for example, the likelihood of coral disease increases from 4 to 89% when they are in contact with plastic^48^. The impacts of large-scale plastic accumulation to both the marine and terrestrial ecosystems on Aldabra are yet to be quantified, for example, how the accumulated waste along the coastline which undergoes extreme degradation by ultraviolet photo-oxidation may interact with run-off and wave action to filter back onto the surrounding coral reefs. The value of clean-up efforts is therefore potentially under-played: they are a vital, if costly, management action, essential for small island nations with coral reef ecosystems, hoping to withstand a suite of impending threats. As such, we believe that in addition to improving waste management, more international funding should be directed towards clean-ups, considering the transboundary nature of the marine plastic litter problem *and* the transboundary nature of the ecosystem services provided by biodiversity-rich islands.

## Methods

### Aldabra atoll

Aldabra Atoll is one of the outer islands of the Seychelles archipelago and lies 1,120 km southwest of Mahé Island (Fig. 2). The closest inhabited land is the northern tip of Madagascar, 407 km to the south, while Tanzania lies 700 km due west. The atoll is 34 km long and 14 km wide and is comprised of four large coral islands (Picard, Polymnie, Malabar, Grande Terre) separated by narrow channels that enclose a shallow tidal lagoon. A fringing coral reef surrounds the atoll and provides partial protection to ocean-facing beaches, on which large numbers of green turtles nest. The atoll experiences two distinct seasons: the northwest monsoon from November to April which brings calmer seas and monsoon rains; and the strong southeast trade-winds from May to October, which are generally dry^49^. Of all the islands, Grande Terre is the most heavily impacted by marine plastic litter, especially during the southeast season, when the trade-winds blow directly onshore. It is also the most difficult part of the atoll to access; hence while clean-ups are regularly conducted by SIF staff on the island of Picard, the south coast has been accumulating litter since it began to arrive—probably in the 1980s. Fifteen turtle-nesting beaches and areas of tortoise-grazing habitat on the Grande Terre coast were deemed the most critical to clean up, as over many years the litter had accumulated into large piles of compacted trash, which inhibits adult turtles from laying eggs and hinders hatchlings from reaching the sea safely.

### Clean-up details

A team of 12 volunteers comprised the clean-up work-force. The team spent three weeks stationed at three field camps. The camps are situated at the only locations along the coast where a boat can land safely from the ocean side: Dune d’Messe (DdM), Dune Jean-Louis (DJL) and Cinq Cases (CC) (Fig. 2). These provided bases from which two teams could usually complete two four-hour cleaning sessions per day. All litter was placed into sacks or slings (with efforts to remove sand and other water) separated into six categories and weighed at the end of each session to get category totals. The six categories were: (1) *consumer plastic items* (e.g. toothbrushes, cigarette lighters); (2) *plastic packaging material* (e.g. PET bottles, cosmetic bottles); (3) *fishing-related items* (e.g. buoys, nets, fish-aggregating devices (FADS) and ropes); (4) *plastic footwear* (mostly flip-flops); (5) *unidentified plastic fragments* > 1 cm in diameter; (6) *other materials* (e.g. glass). These categories were defined based upon those outlined in the Tangaroa Blue Foundation Marine Debris Identification Manual (https://www.tangaroablue.org/resources/clean-up-data-collection/id-manual/), although we grouped all non-plastic items into the ‘Other’ category and segregated footwear into its own category due to the visible dominance of this type of debris. The full sacks and slings were left above the high-tide line at designated collection points.

The most difficult and potentially dangerous phase of the operation was the transfer of the collected litter from shore to ship and this is only possible on the south-coast of Aldabra in either late November/early December or in March, when the sea is calmest due to the shift in seasons. Small boats ferried sacks of litter from the beaches to a waiting supply ship that was anchored beyond the fringing reef and equipped with a crane to lift slings from the small boats onto its deck. For this phase the volunteer teams were assisted by an experienced staff team from the Aldabra research station and a team from the Seychelles Peoples Defence Forces via the coastguard vessel.

### Estimating amounts of plastic debris and costs of removal

The more frequent clean-ups on Picard Island, coupled with the small number of beaches and the higher elevation of the karst on Malabar means that there is substantially less accumulated waste on the northern islands of the atoll. We therefore focus on Grande Terre and consider that cleaning up Grande Terre would be synonymous with cleaning up the whole atoll. To estimate the total amounts and types of marine plastic litter found in different coastal habitats on Aldabra, we chose twenty locations along the south coast of Grande Terre. At each location, three coastal habitat types were identified: (1) sandy beach above the high-tide line (where green turtles nest); (2) coastal limestone karst; and (3) coastal vegetation, within 5 m of the beach or karst, as the amount of marine plastic litter declines with distance away from the coast. There were a small number of places with extreme accumulations, like blowholes (Fig. S3), that were not surveyed. A single 20 m × 2 m transect was laid out at each location in each habitat type, parallel to the water’s edge. In each transect, we picked up all surface-lying items of rubbish > 1 cm in diameter, including partially buried items. Items < 1 cm in diameter were not collected, as the effort involved in their removal during any large-scale clean-up operation would be prohibitive. All items were sorted into the six categories mentioned above and placed directly into separate collecting bags and weighed using a spring balance at the end of the session. This data was used to estimate how much litter in each category was present in the different habitat types per square metre (Fig. 3). To test whether the amounts of litter in each category varied among habitat types, we fitted a linear mixed effects model (details in supplementary material), where the response variable was the amount of litter removed, and the explanatory variables were litter type and habitat type. Location and transect identity were fitted as nested random effects. The model was fitted using the package *lmer* and *lmerTest* to provide approximate p-values using Satterthwaite’s method in R Studio (version 1.3.959). The data were highly over-dispersed, so we modelled Log(litter mass + 1) which corrected the problem. To scale up from transects to the total litter on Grande Terre island we used the raw data, rather than estimates from the model, which we considered to be a conservative approach (variation associated with location and transect variability is then included in the standard errors of the estimates). The estimate for each litter category in each habitat type was then combined with estimates of the total area of each habitat on Grande Terre (Table S1), taken from Google Earth Pro (details in Supplementary material).

In addition to the transects, we recorded the total amount of litter collected in each of the 40 clean-up sessions, together with the length of the session and the number of people working in it. These numbers were used to estimate the amount of litter collected per person per hour. Once we had estimated the total amount of litter present on Grande Terre, we then estimated the total effort and costs needed to remove all the accumulated litter from Grande Terre, assuming that future teams could work at a similar rate.

We provide a breakdown of costs for the clean-up operation and use these to estimate the costs per day of clean-up (our costs divided by the number of clean-up days) and costs per tonne (our costs divided by the number of tonnes) and the projected costs of a full-island clean-up (costs per tonne multiplied by estimated total island tonnage), (Table 1). The main costs were: transport from Mahé to Aldabra in both directions for a team of 12 people; hiring a cargo vessel for 18 days (four days travel in each direction, plus one week of loading at Aldabra and three days unloading on Mahé) and the sacks and slings used. The staffing costs were based on an average daily-salary rate for the SIF team based on Aldabra. We have assumed that similar methodologies to those described here would be employed in any future clean-up effort of Aldabra or other Seychelles outer island: for example, the cargo vessel was the third largest in Seychelles and it was filled during this expedition, so to remove a larger volume at a single event would require either multiple vessels or multiple trips. The Seychelles Peoples Defence Force team were provided without charge by the Government of Seychelles, but there is no guarantee that they could assist with future expeditions and so this was not included in cost projections, making our estimates of cost conservative. Due to their being no cost data available from other island clean-ups, it is not possible to do more than present our own experience and this may help other managers planning clean-ups within and outside of Seychelles.

### Origin of marine plastic litter

All the plastic litter was moved from Aldabra to a storage facility in Mahé. Once there, we estimated the total volume of plastic and sampled six haphazardly-selected sacks of PET bottles (a total of 470 bottles, approximately 1.2% of total collected) to record the type and origin of all the bottles on which labels remained. For the fishing buoys we recorded the brand and/or origin of 50 buoys, also selected haphazardly. We also recorded the identification codes on each fish aggregation device (FAD); a free-floating human-made raft equipped with floats to ensure buoyancy and a sea anchor mostly built from old fishing net that are deployed to attract schools of fish in purse-seine fisheries, they are remotely monitored with a GPS-tracked buoy that includes an echo sounder unit^50,51^. Using the identification codes we could attempt to track their origins (FADs are usually labelled with the ship code they came from, and we checked this against the list of fishing vessels operating within the region).

### Informed consent

The consent of all persons in photographs for figures was obtained for publication of identifying information/images in an online open-access publication.

## Supplementary information

Supplementary Information 1.

Supplementary Information 2.

Supplementary Information 3.

## Data Availability

The datasets generated during and/or analysed during the current study are available from the corresponding author on reasonable request.
