# Supplementary material for: The costs of removing the unsanctioned import of marine plastic litter to small island states
Source: Sci Rep. 2020 Sep 10;10:14458. doi: 10.1038/s41598-020-71444-6 (PMC7483532; doi:10.1038/s41598-020-71444-6)
Supplement: Supplementary file 1 — Supplementary Information 1. [file 41598_2020_71444_MOESM1_ESM.pdf]

The costs of removing the unsanctioned import of marine plastic litter to small island states

April J Burt[1,2], Jeremy Raguain[2], Cheryl Sanchez[2], Jude Brice[2], Frauke Fleischer-Dogley[2], Rebecca Goldberg[3], Sheena Talma[2], Martyna Syposz[3], Josephine Mahony[4], Jake Letori[2], Christina Quanz[2], Sam Ramkalawan[2], Craig Francourt[2], Ivan Capricieuse[2], Ash Antao[2], Kalsey Belle[2], Thomas Zillhardt[5], Jessica Moumou[2], Marvin Roseline[2], Joel Bonne[2], Ronny Marie[2], Edward Constance[2], Jilani Suleman[2], Lindsay A Turnbull[1,2]*

1. Department of Plant Sciences, Oxford University, South Parks Road, OX1 3RB, UK

2. Seychelles Islands Foundation, La Ciotat Building, Mont Fleuri, Victoria, Mahé, Seychelles

3. Department of Zoology, Oxford University, 1a Mansfield Rd, Oxford, OX1 3SZ, UK

4. Department of Geography, Oxford University, South Parks Road, Oxford OX1 3QY, UK

5. Department of Materials Science, Oxford University, 21 Banbury Rd, Oxford OX2 6HT, UK

***Corresponding author: Lindsay.turnbull@plants.ox.ac.uk**

Supplementary material

Habitat type area calculation methods

To estimate beach area: Area polygons were created in google earth pro for all south facing beaches on the south-coast of Grande Terre island (i.e. all the beaches targeted during the clean-up expedition). The west coast beaches of Grande Terre island were not included in this area total, neither were any beaches from Picard, Malabar or Polymnie islands. These beaches were not included in our overall total because they are under different wind and current conditions and without additional transects on these areas it is not representative to extrapolate to these. These other beaches may not accumulate plastic at the same rate and have been subject to periodic clean-ups by the Aldabra team.

To estimate coastal karst area: At thirty haphazard points along the seaward coastline, the width of the karst was measured using google earth pro, these were averaged to 16.05m (stDev: 4.5m). The total outer (seaward) rim length was calculated also in google earth pro. The total karst area was then calculated by multiplying estimated karst width by outer rim length (52.5 km).

To estimate coastal vegetation area: Although trash did spread further into the vegetation it was most highly concentrated in the first 5 m. We therefore confined our estimated to a width of 5m and multiplied this by the total outer (seaward) rim length to get an area we felt was most representative of the spread of trash into the vegetation.

Table S1: Estimated quantity and composition of accumulated marine plastic litter on the coast of Grande Terre based on surveys of the three dominant coastal habitats.

Terrain Type	Terrain Area m^2	Debris category	Mean kg per m^2	Standard Deviation	N	Standard Error	95% Confidence Interval	Total per category (tonnes)
**Vegetation**	262,300	Consumer	0.014	0.011	20	0.002	0.005	3.7
		Fishing related	0.107	0.161	20	0.036	0.07	28.1
		Plastic shoes	0.097	0.132	20	0.03	0.058	25.4
		Fragments	0.044	0.033	20	0.007	0.014	11.5
		Other Debris	0.021	0.055	20	0.012	0.024	5.5
		Packaging	0.045	0.053	20	0.012	0.023	11.8
**Beach**	62,225	Consumer	0.021	0.028	20	0.006	0.012	1.3
		Fishing related	0.093	0.159	20	0.036	0.07	5.8
		Plastic shoes	0.15	0.12	20	0.027	0.053	9.3
		Fragments	0.05	0.037	20	0.008	0.016	3.1
		Other Debris	0.043	0.107	20	0.024	0.047	2.7
		Packaging	0.043	0.031	20	0.007	0.014	2.7
**Karst**	841,983	Consumer	0.001	0.004	20	0.001	0.002	0.8
		Fishing related	0.463	0.604	20	0.135	0.265	389.8
		Plastic shoes	0.003	0.006	20	0.001	0.003	2.5
		Fragments	0.005	0.012	20	0.003	0.005	4.2
		Other Debris	0.002	0.006	20	0.001	0.002	1.7
		Packaging	0.004	0.011	20	0.003	0.005	3.4
**Total tonnes for Island (95% CI)**								**513.4 (212–814)**

https://www.environmental-research.ox.ac.uk/supervisors/e-j-milner-gulland/

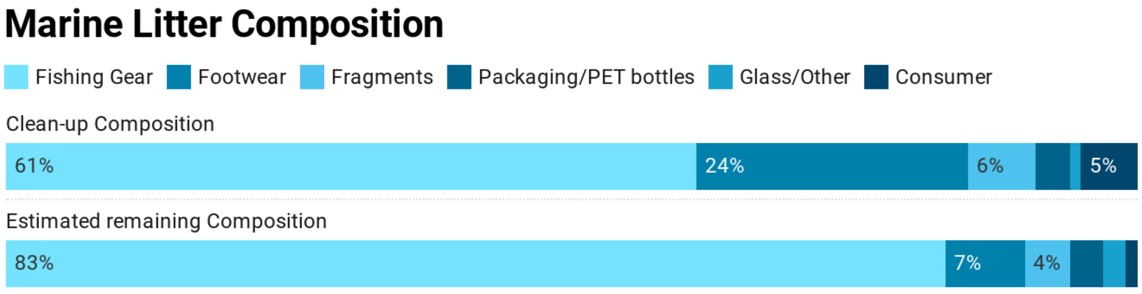

Figure S1: Percentage composition for all marine litter collected during the expedition and the estimated composition of the remaining litter on Aldabra. The difference is due to the expedition focussing on beaches rather than karst, which collects more fishing gear.

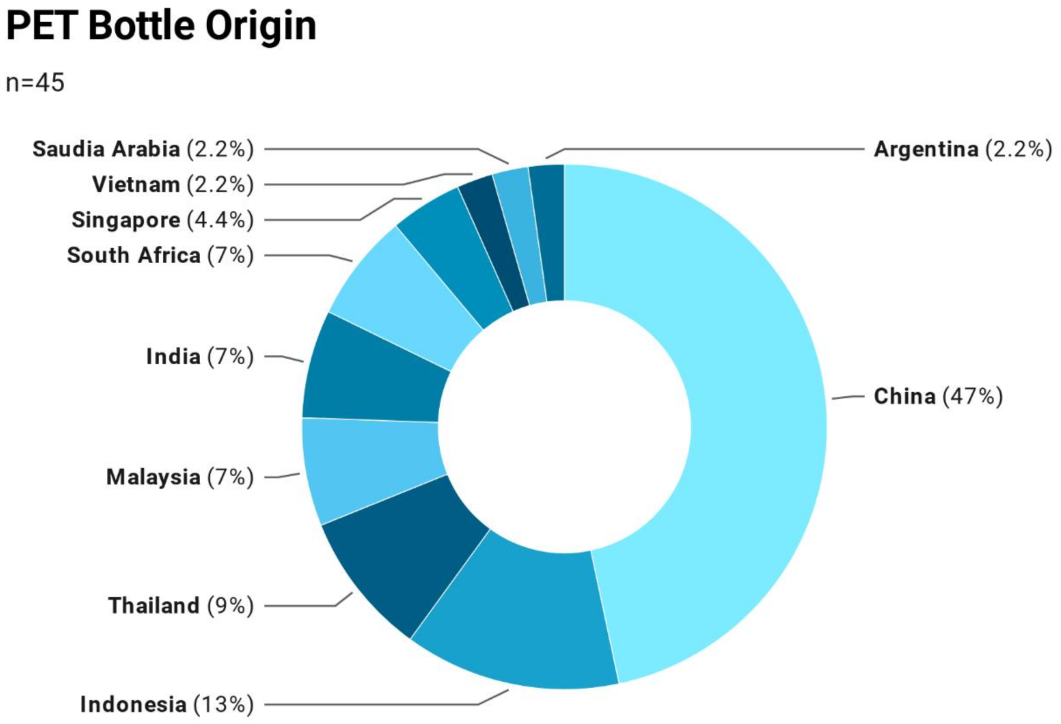

Figure S2: Country of origin for 45 bottles that had labels remaining.

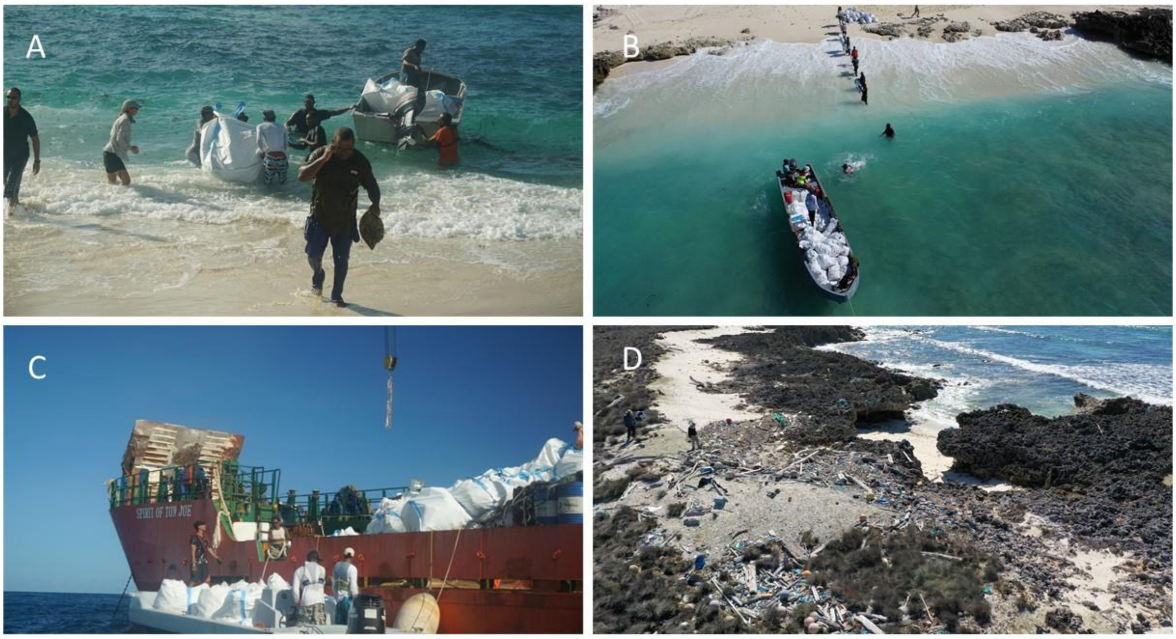

Figure S3: Expedition: A) Loading slings onto small shuttle boat; B) Team loading sacks onto shuttle boat; C) Loading slings onto vessel; D) Karst inlet which trap trash brought by crashing waves.

Informed consent

The consent of all persons in photographs for figures was obtained for publication of identifying information/images in an online open-access publication.

Table S2: Potential solutions for long-term, circular plastics economy in Seychelles.

Material	Proposed solution	End Product
All Plastics -polyethylene terephthalate PET (common for beverage bottles and food packaging), polyethylene HDPE (used in detergents, bleach, and motor oil containers), poly- vinyl chloride PVC (toys, furnishing), polyurethane (beach sandals), and polystyrene PS.	Pyrolysis is the process of decomposing plastic at moderately high temperatures, in the absence of oxygen, to produce an oil-like substance. Significantly, the process is able to handle mixed plastics, negating the need for sorting and separation.	Oil/Fuel
Mostly fishing nets and gear e.g. polypropylene	Plastix Global in Denmark transform used, obsolete and abandoned fishing nets, ropes and post-use rigid plastic that would previously have ended up in the ocean or on landfill, by mechanically recycling them into high-quality raw plastic material: Green Plastic.	Kayaks, furniture, packaging, phone cases, community waste bins, etc.
Fishing gear	Aquafil are a company who turn nets into a material called ECONYL, which companies such as Fourth Element - a dive clothing brand - make swimwear and rash-guards.	Clothing
Flip-flops	TerraCycle is an innovative recycling company that has become a global leader in recycling typically hard-to-recycle waste.	None yet
