# Supplementary material for: The costs of removing the unsanctioned import of marine plastic litter to small island states
Source: Sci Rep. 2020 Sep 10;10:14458. doi: 10.1038/s41598-020-71444-6 (PMC7483532; doi:10.1038/s41598-020-71444-6)
Supplement: Supplementary file 2 — Supplementary Information 2. [file 41598_2020_71444_MOESM2_ESM.pdf]

ACUP_Transect_

April Burt

6/5/2020

Set Up & read in data

```r
rm(list = ls())

## Step 0: Load library----
library(ggplot2)
library(dplyr)
```

```
##
## Attaching package: 'dplyr'

## The following objects are masked from 'package:stats':
##
##     filter, lag

## The following objects are masked from 'package:base':
##
##     intersect, setdiff, setequal, union
```

```r
library(tidyr)
library(lubridate)
```

```
##
## Attaching package: 'lubridate'

## The following objects are masked from 'package:base':
##
##     date, intersect, setdiff, union
```

```r
library(ggfortify)
library(lme4)
```

```
## Loading required package: Matrix

##
## Attaching package: 'Matrix'

## The following objects are masked from 'package:tidyr':
##
##     expand, pack, unpack
```

```r
library(lmerTest)
```

```
##
## Attaching package: 'lmerTest'

## The following object is masked from 'package:lme4':
```

```
##
##     lmer

## The following object is masked from 'package:stats':
##
##     step
```

```
## Step 1: Import the data----
Quantityx <- read.csv("ACUP_Transect_Location.csv")
```

```
str(Quantityx)
```

```
## 'data.frame':    360 obs. of  6 variables:
##  $ Terrain.type           : chr  "Back-beach Vegetation" "Back-beach Vegetation" "Back-beach Vegetati
##  $ Transect.number        : int  44 45 8 14 48 49 11 12 46 47 ...
##  $ Survey.Location        : int  1 2 3 4 5 6 7 8 9 10 ...
##  $ Location..Beach.Number.: num  23 23 24 24 24 24 25 26 26 26 ...
##  $ Type                   : chr  "Consumables..kg." "Consumables..kg." "Consumables..kg." "Consumable
##  $ Weight_kg              : num  1 0.5 1.5 0 0.5 1 1 0.5 0.2 1 ...
```

```
summary(Quantityx)
```

```
##  Terrain.type       Transect.number Survey.Location Location..Beach.Number.
##  Length:360         Min.   : 1.00   Min.   : 1.00   Min.   :23.00
##  Class :character   1st Qu.:15.75   1st Qu.: 5.75   1st Qu.:24.00
##  Mode  :character   Median :30.50   Median :10.50   Median :26.50
##                     Mean   :30.50   Mean   :10.50   Mean   :27.70
##                     3rd Qu.:45.25   3rd Qu.:15.25   3rd Qu.:30.62
##                     Max.   :60.00   Max.   :20.00   Max.   :36.00
##      Type             Weight_kg
##  Length:360         Min.   : 0.000
##  Class :character   1st Qu.: 0.000
##  Mode  :character   Median : 0.500
##                     Mean   : 2.507
##                     3rd Qu.: 2.000
##                     Max.   :70.000
```

Step 1: Fit model

```
mod1. <- lm(Weight_kg~Type*Terrain.type, data=Quantityx)
```

Step 2:Check assumptions

```
autoplot(mod1., smooth.colour = NA)
```

```
## Warning: `arrange_()` is deprecated as of dplyr 0.7.0.
## Please use `arrange()` instead.
## See vignette('programming') for more help
## This warning is displayed once every 8 hours.
## Call `lifecycle::last_warnings()` to see where this warning was generated.

## Warning: Removed 360 row(s) containing missing values (geom_path).

## Warning: Removed 360 row(s) containing missing values (geom_path).
```

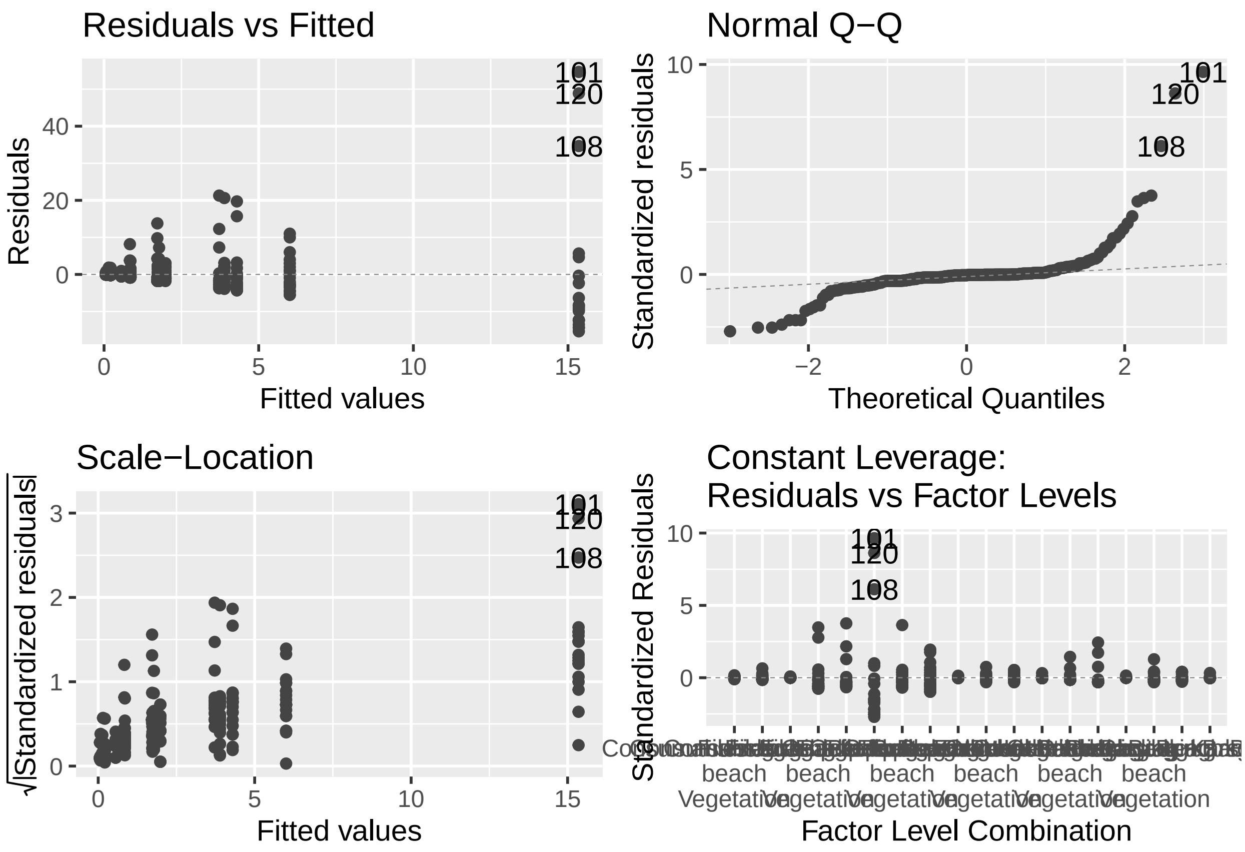

##statistics

```r
anova(mod1.)
```

```
## Analysis of Variance Table
##
## Response: Weight_kg
##                     Df  Sum Sq Mean Sq F value    Pr(>F)
## Type                 5  2302.6  460.53 13.6306 3.846e-12 ***
## Terrain.type         2    18.3    9.13  0.2703    0.7633
## Type:Terrain.type   10  2159.7  215.97  6.3922 4.967e-09 ***
## Residuals          342 11554.9   33.79
## ---
## Signif. codes:  0 '***' 0.001 '**' 0.01 '*' 0.05 '.' 0.1 ' ' 1
```

```r
summary(mod1.)
```

```
##
## Call:
## lm(formula = Weight_kg ~ Type * Terrain.type, data = Quantityx)
##
## Residuals:
##     Min      1Q  Median      3Q     Max
## -15.350  -1.275  -0.210   0.119  54.650
##
## Coefficients:
##                                           Estimate Std. Error t value Pr(>|t|)
## (Intercept)                                 0.5575     1.2997   0.429   0.6682
```

```
## TypeFishing.Gear..k.                        3.7375     1.8381   2.033   0.0428
## TypeFlip.flops..kg.                         3.3325     1.8381   1.813   0.0707
## TypeFragments..kg.                          1.2175     1.8381   0.662   0.5082
## TypeOther.debris..kg.                       0.2775     1.8381   0.151   0.8801
## TypePackaging..kg.                          1.2225     1.8381   0.665   0.5064
## Terrain.typeBeach                           0.2955     1.8381   0.161   0.8724
## Terrain.typeKarst                          -0.5015     1.8381  -0.273   0.7851
## TypeFishing.Gear..k.:Terrain.typeBeach     -0.8680     2.5995  -0.334   0.7386
## TypeFlip.flops..kg.:Terrain.typeBeach       1.8195     2.5995   0.700   0.4844
## TypeFragments..kg.:Terrain.typeBeach       -0.0855     2.5995  -0.033   0.9738
## TypeOther.debris..kg.:Terrain.typeBeach     0.5920     2.5995   0.228   0.8200
## TypePackaging..kg.:Terrain.typeBeach       -0.3405     2.5995  -0.131   0.8959
## TypeFishing.Gear..k.:Terrain.typeKarst     11.5565     2.5995   4.446 1.19e-05
## TypeFlip.flops..kg.:Terrain.typeKarst      -3.2575     2.5995  -1.253   0.2110
## TypeFragments..kg.:Terrain.typeKarst       -1.0630     2.5995  -0.409   0.6828
## TypeOther.debris..kg.:Terrain.typeKarst    -0.2585     2.5995  -0.099   0.9208
## TypePackaging..kg.:Terrain.typeKarst       -1.1260     2.5995  -0.433   0.6652
##
## (Intercept)
## TypeFishing.Gear..k.                      *
## TypeFlip.flops..kg.                       .
## TypeFragments..kg.
## TypeOther.debris..kg.
## TypePackaging..kg.
## Terrain.typeBeach
## Terrain.typeKarst
## TypeFishing.Gear..k.:Terrain.typeBeach
## TypeFlip.flops..kg.:Terrain.typeBeach
## TypeFragments..kg.:Terrain.typeBeach
## TypeOther.debris..kg.:Terrain.typeBeach
## TypePackaging..kg.:Terrain.typeBeach
## TypeFishing.Gear..k.:Terrain.typeKarst    ***
## TypeFlip.flops..kg.:Terrain.typeKarst
## TypeFragments..kg.:Terrain.typeKarst
## TypeOther.debris..kg.:Terrain.typeKarst
## TypePackaging..kg.:Terrain.typeKarst
## ---
## Signif. codes:  0 '***' 0.001 '**' 0.01 '*' 0.05 '.' 0.1 ' ' 1
##
## Residual standard error: 5.813 on 342 degrees of freedom
## Multiple R-squared:  0.2794, Adjusted R-squared:  0.2436
## F-statistic: 7.801 on 17 and 342 DF,  p-value: < 2.2e-16
```

MEM

```
mem1 <- lmer(log(Weight_kg+1) ~ Type * Terrain.type + (1|Survey.Location/Transect.number), data = Quant
```

```
anova(mem1, type=1)
```

```
## Type I Analysis of Variance Table with Satterthwaite's method
##                    Sum Sq Mean Sq NumDF DenDF F value    Pr(>F)
## Type               63.101 12.6202     5   285  42.058 < 2.2e-16 ***
## Terrain.type        8.727  4.3633     2    38  14.541 2.043e-05 ***
## Type:Terrain.type  44.897  4.4897    10   285  14.962 < 2.2e-16 ***
## ---
```

```
## Signif. codes:  0 '***' 0.001 '**' 0.01 '*' 0.05 '.' 0.1 ' ' 1
```

```r
plot(mem1, smooth.colour = NA)
```

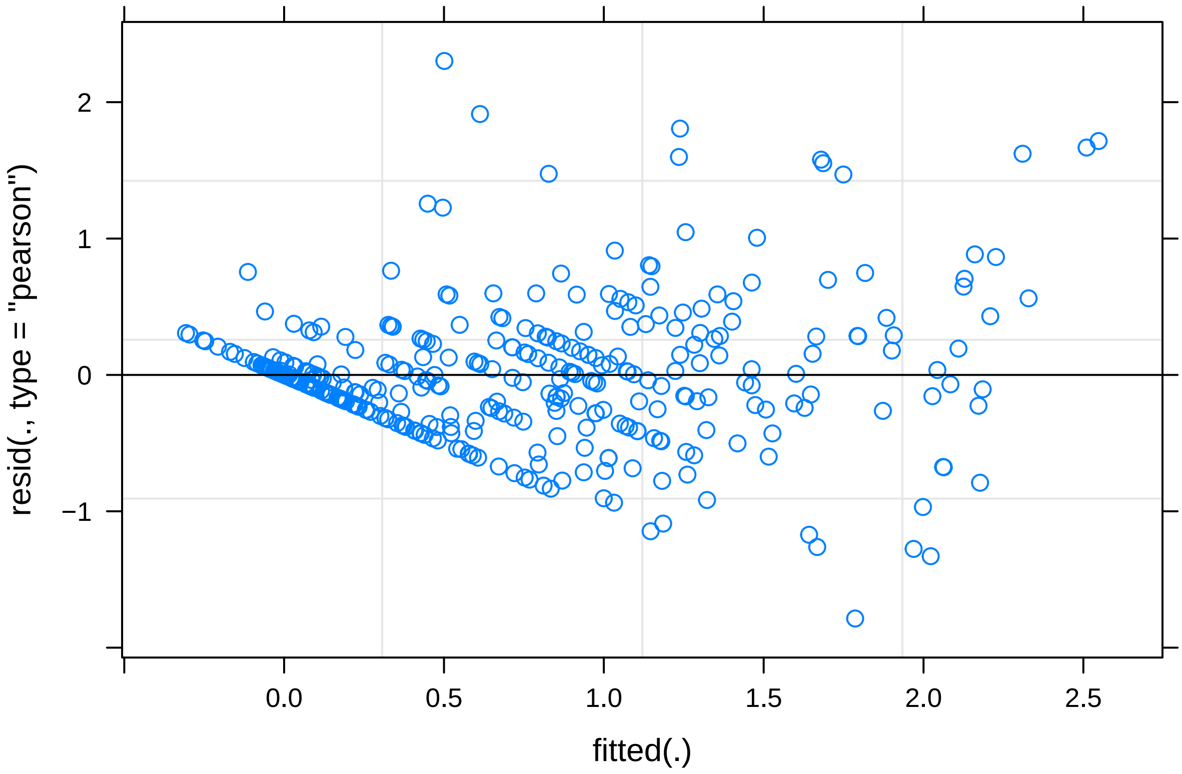


```r
summary(mem1)
```

```
## Linear mixed model fit by REML. t-tests use Satterthwaite's method [
## lmerModLmerTest]
## Formula:
## log(Weight_kg + 1) ~ Type * Terrain.type + (1 | Survey.Location/Transect.number)
##    Data: Quantityx
##
## REML criterion at convergence: 663.4
##
## Scaled residuals:
##     Min      1Q  Median      3Q     Max
## -3.2607 -0.4608 -0.0507  0.3640  4.2026
##
## Random effects:
##  Groups                          Name        Variance Std.Dev.
##  Transect.number:Survey.Location (Intercept) 0.04480  0.2117
##  Survey.Location                 (Intercept) 0.03489  0.1868
##  Residual                                    0.30007  0.5478
## Number of obs: 360, groups:
## Transect.number:Survey.Location, 60; Survey.Location, 20
##
## Fixed effects:
##                                        Estimate Std. Error         df
## (Intercept)                            0.405202   0.137797 258.803078
## TypeFishing.Gear..k.                   0.812143   0.173225 285.000032
```

```
## TypeFlip.flops..kg.                       0.856281   0.173225 285.000032
## TypeFragments..kg.                        0.525402   0.173225 285.000032
## TypeOther.debris..kg.                    -0.109581   0.173225 285.000032
## TypePackaging..kg.                        0.425910   0.173225 285.000032
## Terrain.typeBeach                         0.089830   0.185707 260.839918
## Terrain.typeKarst                        -0.358900   0.185707 260.839918
## TypeFishing.Gear..k.:Terrain.typeBeach   -0.243075   0.244977 285.000033
## TypeFlip.flops..kg.:Terrain.typeBeach     0.362086   0.244977 285.000033
## TypeFragments..kg.:Terrain.typeBeach     -0.034335   0.244977 285.000033
## TypeOther.debris..kg.:Terrain.typeBeach   0.033644   0.244977 285.000033
## TypePackaging..kg.:Terrain.typeBeach     -0.008824   0.244977 285.000033
## TypeFishing.Gear..k.:Terrain.typeKarst    1.280958   0.244977 285.000033
## TypeFlip.flops..kg.:Terrain.typeKarst    -0.796011   0.244977 285.000033
## TypeFragments..kg.:Terrain.typeKarst     -0.425590   0.244977 285.000033
## TypeOther.debris..kg.:Terrain.typeKarst   0.120411   0.244977 285.000033
## TypePackaging..kg.:Terrain.typeKarst     -0.372279   0.244977 285.000033
##                                         t value Pr(>|t|)
## (Intercept)                               2.941  0.00357 **
## TypeFishing.Gear..k.                      4.688 4.27e-06 ***
## TypeFlip.flops..kg.                       4.943 1.31e-06 ***
## TypeFragments..kg.                        3.033  0.00264 **
## TypeOther.debris..kg.                    -0.633  0.52750
## TypePackaging..kg.                        2.459  0.01454 *
## Terrain.typeBeach                         0.484  0.62899
## Terrain.typeKarst                        -1.933  0.05437 .
## TypeFishing.Gear..k.:Terrain.typeBeach   -0.992  0.32192
## TypeFlip.flops..kg.:Terrain.typeBeach     1.478  0.14050
## TypeFragments..kg.:Terrain.typeBeach     -0.140  0.88864
## TypeOther.debris..kg.:Terrain.typeBeach   0.137  0.89086
## TypePackaging..kg.:Terrain.typeBeach     -0.036  0.97129
## TypeFishing.Gear..k.:Terrain.typeKarst    5.229 3.31e-07 ***
## TypeFlip.flops..kg.:Terrain.typeKarst    -3.249  0.00130 **
## TypeFragments..kg.:Terrain.typeKarst     -1.737  0.08342 .
## TypeOther.debris..kg.:Terrain.typeKarst   0.492  0.62344
## TypePackaging..kg.:Terrain.typeKarst     -1.520  0.12971
## ---
## Signif. codes:  0 '***' 0.001 '**' 0.01 '*' 0.05 '.' 0.1 ' ' 1
##
## Correlation matrix not shown by default, as p = 18 > 12.
## Use print(x, correlation=TRUE)  or
##     vcov(x)        if you need it
```
